# Survival Predictors and Clinical Outcomes in Patients Undergoing Venoarterial ECMO: A 7-Year Retrospective Study

**DOI:** 10.1155/ccrp/5588093

**Published:** 2025-08-22

**Authors:** Thavat Chanchayanon, Mantana Saetang, Sutthiphat Wangpholpattanasiri, Ratikorn Boonchai, Pongsanae Duangpakdee

**Affiliations:** ^1^Department of Anesthesiology, Faculty of Medicine, Prince of Songkla University, Hat-Yai, Songkhla 90110, Thailand; ^2^Department of Surgery, Faculty of Medicine, Prince of Songkla University, Songkhla 90110, Thailand

**Keywords:** complications, extracorporeal membrane oxygenation, mortality, risk stratification, survival predictors, VA-ECMO

## Abstract

**Purpose:** Venoarterial extracorporeal membrane oxygenation (VA-ECMO) is a life-saving intervention for refractory cardiopulmonary failure. Identifying factors associated with survival is essential for optimizing patient selection and management. In this study, we aimed to identify VA-ECMO survival predictors and evaluate the associated complications, costs, and outcomes.

**Methods:** A retrospective analysis was conducted on data from 123 adult patients who underwent VA-ECMO at the Songklanagarind Hospital between 2017 and 2023. Clinical characteristics, ECMO-related complications, hospital expenses, and survival outcomes were analyzed. Univariate and multivariate logistic regression analyses were used to determine independent predictors of survival.

**Results:** Fifty (40.7%) patients survived until hospital discharge. Compared to central VA-ECMO, peripheral VA-ECMO was significantly associated with improved survival (adjusted OR: 26.44, 95% CI: 1.95–358.7, *p* = 0.014). Preexisting liver dysfunction (adjusted OR: 0.27, 95% CI: 0.09–0.79, *p* = 0.016) and renal dysfunction (adjusted OR: 0.29, 95% CI: 0.1–0.85, *p* = 0.023) were independent mortality predictors. Survival odds were significantly lower in patients with American Society of Anesthesiologists (ASA) Class 5 (adjusted OR: 0.07, 95% CI: 0.01–0.67, *p* = 0.022). Neurological complications were more common in nonsurvivors than in survivors (41.1% vs. 18%, *p* = 0.012). Survivors had significantly higher total hospital costs (997,563.5 vs. 696,191 THB, *p* = 0.004) and longer hospital stays (28.5 vs. 3 days, *p* < 0.001). The multivariate model demonstrated strong predictive performance, with an area under the curve of 0.85.

**Conclusions:** ECMO cannulation strategy, preexisting liver and renal dysfunction, and ASA classification were key factors associated with survival. Peripheral VA-ECMO was associated with better outcomes, and organ dysfunction significantly increased the mortality risk.

## 1. Introduction

Venoarterial extracorporeal membrane oxygenation (VA-ECMO) is a mechanical circulatory support system used in patients with severe cardiac and respiratory failure refractory to conventional treatments [1]. In patients with acute, severe, yet potentially reversible cardiac injuries, such as myocarditis and myocardial ischemia, VA-ECMO may provide a bridge to recovery. In patients with acute decompensated chronic cardiac failure or massive myocardial infarction, VA-ECMO may be used as a bridge to destination therapy, such as the insertion of a durable ventricular assist device or cardiac transplantation [2]. All VA-ECMO circuits consist of a venous (inflow and drainage) cannula, pump, oxygenator, and arterial (outflow and return) cannula. VA-ECMO can be performed via peripheral or central access [1]. Despite technological advancements and increasing utilization, VA-ECMO is associated with significant morbidity and mortality [3, 4], with survival rates varying between 27% and 59% depending on the patient population and indications [5–8].

Several factors have been identified as predictors of survival in patients undergoing VA-ECMO, including patient demographics, comorbid conditions, hemodynamic parameters, and ECMO-related complications [9–11]. Moreover, VA-ECMO is resource-intensive, with reported costs reaching approximately 145,580 USD per patient in the Netherlands [12] and 1,064,265 THB (35,475.5 USD) per patient in Thailand [13]. The high financial burden associated with ECMO further underscores the need for effective patient selection and resource allocation to optimize clinical and economic outcomes.

Despite these insights, there remains a lack of consensus on the most critical prognostic factors influencing patient selection, management strategies, and resource allocation. Most studies investigating VA-ECMO outcomes have been conducted in Western populations, with limited data available from Southeast Asia. Given the potential differences in patient demographics, healthcare infrastructure, and ECMO management protocols, further research is warranted to investigate the determinants of survival in Southeast Asian populations.

In this study, we aimed to evaluate factors associated with the survival of patients undergoing VA-ECMO at a tertiary care hospital in Thailand. By adopting a retrospective observational design, we examined key prognostic indicators, including comorbidities, ECMO strategies, and post-ECMO complications. Moreover, the financial costs of VA-ECMO therapy were assessed to provide a comprehensive evaluation of both clinical and economic outcomes. By identifying the predictors of mortality and analyzing the complications and healthcare costs, this study contributes valuable insights into clinical decision-making for improving patient outcomes in VA-ECMO therapy.

## 2. Materials and Methods

### 2.1. Study Design and Setting

This retrospective cohort study was conducted at Songklanagarind Hospital, a tertiary care referral center in Thailand. Data were collected from the electronic medical records of the hospital and the perfusionist ECMO database between January 2017 and December 2023.

### 2.2. Study Population

The study included adult patients (≥ 15 years) who received VA-ECMO support during the study period. Patients with incomplete medical records or those for whom full ECMO flow could not be established were excluded from the study.

### 2.3. Data Collection

Patient demographics, clinical characteristics, comorbidities, data on ECMO indications, and perioperative management details were extracted from the hospital records. Complications including acute kidney injury (AKI), bleeding, infection, neurological events, and vascular complications were recorded. Economic data, including total hospital expenses, medication costs, blood product utilization, and procedural expenses, were also analyzed. The endpoint for data collection was hospital discharge (survival) or in-hospital death.

### 2.4. Variables and Operational Definitions

The choice between central and peripheral VA-ECMO was determined by the clinical team based on the patient condition and surgical context. Central VA-ECMO was primarily used in patients with postcardiotomy cardiogenic shock following open cardiac surgery or in patients for whom peripheral cannulation was not feasible due to vascular anatomy, severe peripheral arterial disease, or prior vascular surgery. Meanwhile, peripheral VA-ECMO was generally preferred for patients without postcardiotomy cardiogenic shock or those who underwent extracorporeal cardiopulmonary resuscitation (ECPR) when rapid initiation at the bedside was required.

Regarding complications, renal dysfunction was defined as serum creatinine > 1.5 mg/dL, with or without the need for renal replacement therapy. Liver dysfunction refers to the presence of abnormal liver function tests (aspartate aminotransferase or alanine aminotransferase > 3 times the upper limit of normal). Central nervous system (CNS) dysfunction was defined as dysfunctions caused by conditions such as neurotrauma, stroke, encephalopathy, seizure, or epileptic syndrome, or other conditions that led to a Glasgow Coma Scale score of < 8. Respiratory failure was defined as the requirement for positive pressure mechanical ventilatory support or with a partial pressure of oxygen/fraction of inspired oxygen < 200 mmHg. Bleeding refers to a clinically significant hemorrhage occurring during ECMO support, as documented in the hospital discharge summary.

### 2.5. Outcomes

The primary outcome was in-hospital survival in patients undergoing VA-ECMO at Songklanagarind Hospital. In-hospital survival rate was defined as the proportion of VA-ECMO patients who were discharged alive from the hospital compared to all VA-ECMO patients. As we collected patient data until hospital discharge (survival) or in-hospital death, patients who died after hospital discharge were classified as survivors. Secondary outcomes included admission expenses, complications associated with VA-ECMO, and duration of ECMO support in survivors and nonsurvivors.

### 2.6. Sample Size Calculation

The sample size was determined using a formula derived from cohort study methodology, incorporating the proportions of the outcome of interest in both the exposed and unexposed groups. The primary outcome was in-hospital survival, with exposure and nonexposure corresponding to the presence or absence of factors associated with survival, respectively. As demonstrated in a cohort study by Schmidt et al. [7], pre-ECMO cardiac arrest is a significant prognostic factor that influences survival outcomes. The reported prevalence of pre-ECMO cardiac arrest is 28.7% (*p*_1_) among survivors and 66.0% (*p*_2_) among nonsurvivors, based on empirical data from a previous study. These proportions were used to calculate the required sample size for the analysis, as follows:

(1) $$\begin{array}{c} n_{1}={\left[\frac{z_{1-\left(\alpha /2\right)}\sqrt{\overset{¯}{p}\overset{¯}{q}\left(1+\left(1/r\right)\right)}+z_{1-\beta }\sqrt{p_{1}q_{1}+\left(\left(p_{2}q_{2}\right)/r\right)}}{\Delta }\right]}^{2}, \end{array}$$

(2) $$\begin{array}{c} r=\frac{n_{2}}{n_{1}},\;q_{1}=1-p_{1},\;q_{2}=1-p_{2}, \end{array}$$

(3) $$\begin{array}{c} \overset{¯}{p}=\frac{p_{1}+p_{2}r}{1+r},\;\overset{¯}{q}=1-\overset{¯}{p}. \end{array}$$

Based on an estimated proportion of pre-ECMO cardiac arrest in survivors (*p*_1_) of 0.287 and estimated proportion of pre-ECMO cardiac arrest in nonsurvivors (*p*_2_) of 0.660, a Type I error (α) of 0.05 and Type II error (β) of 0.10, and a ratio of 1:2, the sample sizes for the exposed and nonexposed groups were 27 and 54, respectively, corresponding to a total of 81 patients. Assuming 10% incomplete data, a total sample size of 90 was deemed adequate to identify one risk factor. Given that the primary analysis was multivariable, we also evaluated adequacy using the events-per-variable (EPV) principle. The final logistic regression model included 6 variables, with 73 mortality events, yielding an EPV of approximately 12.2, which meets the recommended threshold (≥ 10) for avoiding overfitting.

### 2.7. Statistical Analysis

Continuous variables were assessed for normality and are presented as mean ± standard deviation (SD) for normally distributed data or median (interquartile range [IQR]) for non-normally distributed data. Categorical variables were expressed as frequencies and percentages. Univariate analysis was performed using the chi-square or Fisher's exact test for categorical variables and the Student's *t*-test or Wilcoxon rank-sum test for continuous variables.

Candidate predictors for inclusion in the multivariable logistic regression model were selected in two steps: (1) variables with *p* < 0.2 in univariate analysis were considered eligible, and (2) a backward stepwise elimination procedure based on the Akaike information criterion (AIC) was applied. Variables with *p* < 0.05 in the final model were retained as independent predictors.

Collinearity was assessed using the variance inflation factor, with all values < 2, indicating no significant multicollinearity. Model performance was internally validated using bootstrap resampling (200 iterations). Discrimination was assessed using the area under the receiver operating characteristic (ROC) curve (AUC). Calibration was evaluated using bootstrap-corrected calibration curves and the Brier score. The scaled Brier score was calculated to assess the model's improvement over a noninformative model. Statistical significance was set at *p* < 0.05. All analyses were performed using R statistical software (Version 4.4.1) with the rms and pROC packages.

### 2.8. Ethical Considerations

The study protocol was reviewed and approved by the Human Research Ethics Committee of the Faculty of Medicine at the Prince of Songkla University (REC. 66-136-8-1). The requirement for informed consent was waived due to the retrospective design of the study.

## 3. Results

**Figure 1:**
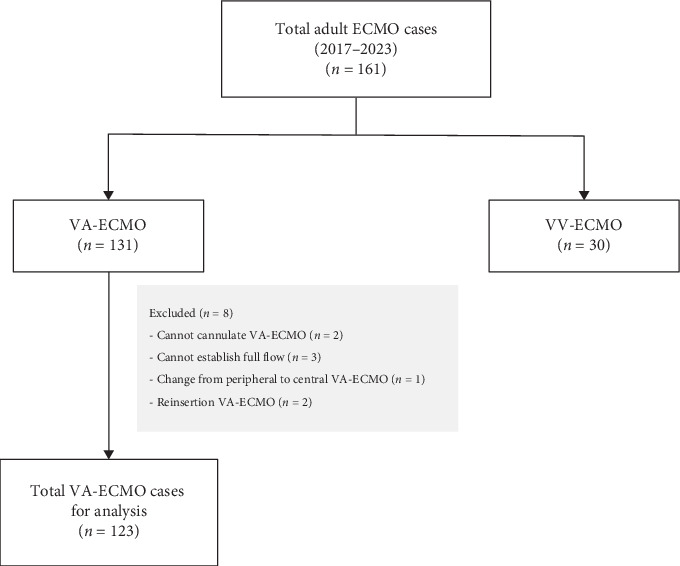
Flow diagram illustrating patient selection and exclusion criteria for VA-ECMO cases. ECMO: extracorporeal membrane oxygenation; VA: venoarterial; VV: venovenous.

A total of 161 adult patients underwent ECMO over a 7-year period (2017–2023). Of these, 131 patients underwent VA-ECMO (Figure 1). Eight patients were excluded based on the following criteria: inability to achieve cannulation (*n* = 2), failure to establish full ECMO flow (*n* = 3), conversion from peripheral to central cannulation (*n* = 1), and reinsertion of ECMO (*n* = 2). Therefore, the final analytical cohort comprised 123 patients who underwent VA-ECMO. The in-hospital survival rate was 40.7%.

### 3.1. Baseline Characteristics and Variables

Peripheral VA-ECMO accounted for 91.9% of the ECMO cases. This approach was used in 96% of survivors and 89% of nonsurvivors (*p* = 0.198). Central VA-ECMO was used in 8.1% of cases, with a higher proportion among nonsurvivors (11%) than among survivors (4%) (*p* = 0.198). General anesthesia was more common than local anesthesia in both groups, with 92% of survivors and 78.1% of nonsurvivors undergoing general anesthesia (*p* = 0.071). The median age of survivors was 56 years (IQR: 39–63 years), compared to 59 years (IQR: 45–68 years) for nonsurvivors (*p* = 0.099). Age > 65 years was significantly associated with nonsurvival (37% vs. 18%, *p* = 0.038). Survivors exhibited higher systolic (116.7 vs. 98.5 mmHg, *p* = 0.002) and diastolic blood pressures (60 vs. 55 mmHg, *p* = 0.018). A mean arterial pressure (MAP) < 80 mmHg was observed more frequently among nonsurvivors (74.1%) than survivors (52.3%), whereas MAP ≥ 80 mmHg was observed in 47.7% of survivors and 25.9% of nonsurvivors (*p* = 0.038). No significant differences were observed in the prevalence of comorbidities (Table 1).

Organ dysfunction was significantly more prevalent in nonsurvivors than in survivors and included liver dysfunction (57.5% vs. 26%, *p* = 0.001), renal dysfunction (56.2% vs. 30%, *p* = 0.007), respiratory dysfunction (80.8% vs. 62%, *p* = 0.035), and CNS dysfunction (56.2% vs. 36%, *p* = 0.044). The use of high-dose vasoactive support (90.4% vs. 68%, *p* = 0.004) and an intraaortic balloon pump (31.5% vs. 14%, *p* = 0.045) were significantly associated with nonsurvival rates. Survivors exhibited significantly higher bicarbonate (19.2 vs. 15.7 mmol/L, *p* = 0.001), lower lactate (3.8 vs. 8.9 mmol/L, *p* = 0.017), and higher pH (7.3 vs. 7.2, *p* = 0.042) levels (Table 1).

Cardiac arrest–related variables were analyzed in a subgroup of 49 patients (17 survivors and 32 nonsurvivors) (Table 2). The most frequently observed initial rhythm among survivors was ventricular fibrillation (40%), whereas pulseless electrical activity was predominant among nonsurvivors (46.7%). All survivors and 93.8% of nonsurvivors experienced a witnessed cardiac arrest (*p* = 0.537). The median no-flow time (time from cardiac arrest to CPR) was 0 min for all patients (*p* = 0.709). The low-flow time (time from arrest to ECMO flow) was shorter in survivors than in nonsurvivors (52.3 vs. 60.3 min; *p* = 0.381), although this difference was not significant. In addition, a higher proportion of survivors achieved ROSC than nonsurvivors (93.8% vs. 75%, *p* = 0.238).

Multivariate analysis identified several independent predictors of survival (Table 3). Peripheral VA-ECMO was strongly associated with increased survival likelihood compared to central VA-ECMO (adjusted odds ratio [OR]: 26.44, 95% confidence interval [CI]: 1.95–358.7, *p* = 0.014). Liver dysfunction (adjusted OR: 0.27, 95% CI: 0.09–0.79, *p* = 0.016) and renal dysfunction (adjusted OR: 0.29, 95% CI: 0.1–0.85, *p* = 0.023) were significant predictors of mortality. The American Society of Anesthesiologists (ASA) classification was also a critical determinant, with patients in ASA Class 5 having significantly lower odds of survival than those in ASA Class ≤ 3 (adjusted OR: 0.07, 95% CI: 0.01–0.67, *p* = 0.022).

The logistic regression model was further evaluated for its ability to differentiate between survivors and nonsurvivors using ROC curve analysis. The model demonstrated an AUC of 0.85, indicating good predictive performance. Model calibration, assessed using a bootstrap-corrected calibration curve, showed good agreement between predicted and observed outcomes (Brier score = 0.156; scaled Brier score = 0.363).

### 3.2. Complications Related to VA-ECMO

Table 4 shows the complications associated with VA-ECMO. A higher proportion of nonsurvivors experienced AKI (64.4%) than survivors (46%), although the difference was not significant (*p* = 0.066). Bleeding was reported in 41.1% of nonsurvivors and 24% of survivors (*p* = 0.077). Infection occurred almost equally in both groups (56% of survivors and 56.2% of nonsurvivors), with no significant difference (*p* = 1). Neurological complications were significantly more common in nonsurvivors (41.1%) than in survivors (18%; *p* = 0.012). Coagulopathy, cardiac tamponade, limb ischemia, and bowel ischemia showed varying frequencies, but the differences observed were not significant. Rare events such as liver infarction, left ventricular distension, and Harlequin syndrome were observed only in nonsurvivors.

### 3.3. Expenses and Other Outcomes

**Figure 2:**
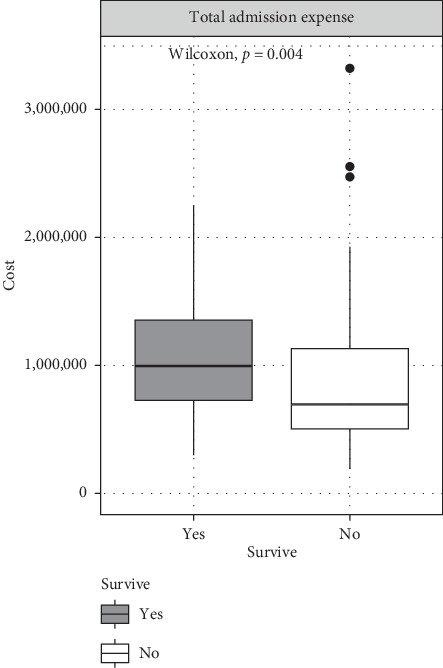
Box plot of total admission expenses.

**Figure 3:**
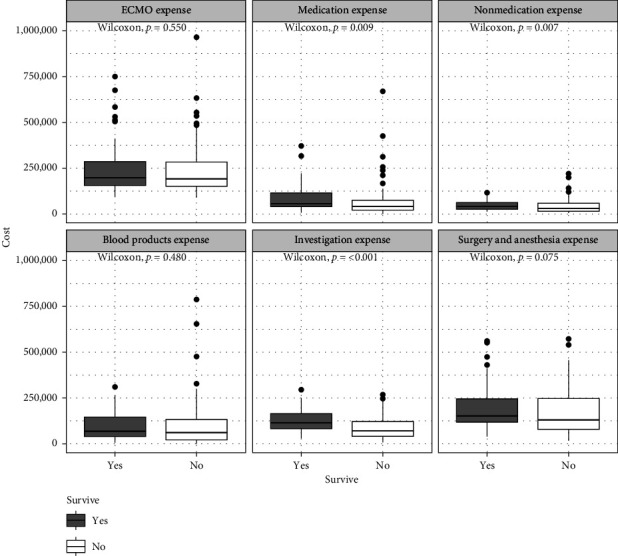
Box plot of admission expenses by category.

The total hospital admission costs were significantly higher in survivors, with a median expense of 997,563.5 (IQR: 728,416.5–1,350,748.8) THB compared to 696,191 (IQR: 504,406–1,124,993) THB in nonsurvivors (*p* = 0.004) (Table 5) (Figure 2). Survivors incurred significantly higher expenses related to medications (56,844.5 THB vs. 42,070 THB, *p* = 0.009), nonmedication hospital resources (42,593 THB vs. 29,736 THB, *p* = 0.007), and diagnostic investigations (119,407.5 THB vs. 69,725 THB, *p* < 0.001). However, no significant differences were noted between survivors and nonsurvivors in ECMO expenses, blood product costs, and surgical- and anesthesia-related expenses (Figure 3).

Survivors had a significantly longer median hospital stay (28.5 [18–46.5] days versus 3 [1–9] days, *p* < 0.001). However, no significant difference existed in the median duration of ECMO support between survivors (3.5 days) and nonsurvivors (3 days) (*p* = 0.145) (Table 5).

## 4. Discussion

We evaluated factors associated with survival in patients undergoing VA-ECMO at a tertiary care center in Thailand. These findings highlight the impact of pre-ECMO organ dysfunction, ASA classification, and ECMO cannulation strategy on survival outcomes. In addition, the differences in complications and healthcare costs between survivors and nonsurvivors provide valuable insights into optimizing patient selection and resource allocation.

In this study, the survival rate of patients undergoing VA-ECMO was 40.7%, which is consistent with reported survival rates ranging from 27% to 59% [6–9]. This rate is slightly lower than the global survival rate of 47% reported in the Extracorporeal Life Support Organization (ELSO) registry [8]. Among patients undergoing ECPR, the in-hospital survival rate was 34.7%, which is consistent with the 31% survival rate reported in the ELSO registry [8]. Although caseload variations, ECMO indications, and institutional protocols may contribute to these differences, our findings highlight the importance of careful pre-ECMO assessment to improve outcomes.

### 4.1. Key Predictors of Survival

Peripheral VA-ECMO was significantly associated with improved survival compared to central VA-ECMO (adjusted OR: 26.44, 95% CI: 1.95–358.7, *p* = 0.014). This finding is consistent with those of previous studies [14–17], suggesting that peripheral cannulation is associated with lower surgical risk, less bleeding, and less infection, potentially contributing to better patient outcomes. Conversely, central VA-ECMO, often employed in postcardiotomy cases, is frequently associated with severe cardiac dysfunction and a higher risk of complications [14–16, 18], which may explain the lower survival rates.

Preexisting liver and renal dysfunction were strong, independent predictors of mortality in the present study. Patients with liver dysfunction had a 73% lower likelihood of survival (adjusted OR: 0.27, 95% CI: 0.09–0.79, *p* = 0.016), whereas renal dysfunction was associated with a 71% reduction in survival odds (adjusted OR: 0.29, 95% CI: 0.1–0.85, *p* = 0.023). These findings are consistent with those of previous studies demonstrating that multiorgan dysfunction before ECMO initiation significantly affects outcomes [7, 19]. Kozakov et al. [19] found that liver dysfunction was the strongest predictor of mortality, with an OR of 2.5. Similarly, Veyret et al. [20] reported that the need for renal replacement therapy is a significant predictor of early mortality in patients undergoing ECMO and may play a role in guiding decisions regarding VA-ECMO initiation. Hepatic and renal impairment likely reflects underlying circulatory failure and metabolic derangements, which reduce the likelihood of recovery despite ECMO support.

ASA classification was also found to be an important predictor, with patients in ASA Class 5 exhibiting significantly lower survival rates than those in ASA Class ≤ 3 (adjusted OR: 0.07, 95% CI: 0.01–0.67, *p* = 0.022). The ASA classification, a widely used preoperative risk stratification tool, has been correlated with postoperative morbidity and mortality in various surgical populations [21]. However, its predictive value in patients undergoing VA-ECMO remains unclear. Our findings suggest that a high ASA classification may serve as an indicator of reduced physiological reserve, thereby influencing survival. Patients with higher ASA scores often present with preexisting organ dysfunction [22], poor baseline functional status, and limited physiological reserve, which may impair their ability to tolerate the metabolic and inflammatory stress induced by VA-ECMO. In addition, these patients are more prone to prolonged ICU stays, increased risk of infections, and a higher incidence of complications, such as bleeding and thrombosis, all of which negatively impact survival. The strong association between ASA classification and mortality highlights its utility in risk stratification and patient selection for VA-ECMO.

### 4.2. ECPR Group

In the ECPR subgroup, with a survival rate of 34.7%, all survivors and 93.8% of nonsurvivors experienced a witnessed cardiac arrest. The no-flow time was 0 min for all patients, while the low-flow time was shorter in survivors (52.3 vs. 60.3 min), although this difference was not significant. According to the ELSO guidelines [23], the ECPR inclusion criteria were a witnessed arrest, a no-flow interval < 5 min, and a low-flow interval < 60 min. Our hospital adhered to these criteria, with nearly all arrests witnessed, no-flow time, and a mean low-flow time of 57.5 min.

### 4.3. Complications Related to VA-ECMO

ECMO-related complications were prevalent in this study, with AKI (56.9%), infection (56.1%), and bleeding (34.1%) being the most common. These findings are consistent with data from the ELSO registry, which reported major complications such as renal failure (48.9%) and bleeding (26.4%) [17]. Notably, neurological complications were significantly more frequent in nonsurvivors (41.1% vs. 18%, *p* = 0.012). Previous studies have identified CNS infarction (4%) and hemorrhage (2%) as significant conditions associated with poor prognosis, often resulting in fatal outcomes [24]. The most devastating complication is intracranial hemorrhage, which is associated with a poor prognosis and is usually fatal [24, 25]. These complications are widely recognized as key contributors to poor neurological and overall survival outcomes.

### 4.4. Expenses and Other Outcomes

ECMO is expensive and resource-intensive. In this study, survivors had significantly higher total admission expenses (997,563.5 THB ≈ 29,479.27 USD). The financial burden of VA-ECMO reported in this study is consistent with previous international cost estimates. In Thailand, the total admission expense for patients undergoing VA-ECMO is approximately 1,064,265 THB (35,475.5 USD) per patient [13], which is comparable to the reported cost of 145,580 USD per patient in the Netherlands [12]. A separate study conducted in Thailand reported different outcomes [13], with nonsurvivors incurring higher admission costs than survivors (9,298,700 vs. 1,483,232 THB). This discrepancy may be attributed to the longer duration of ECMO in nonsurvivors than in the present study (5.47 days in nonsurvivors vs. 8.74 days in survivors). Considering the similar ECMO duration (3 vs. 3.5 days) and notably different times to discharge (28 days) and death (3 days) in the present study, it can be inferred that the higher admission costs in survivors were primarily attributable to expenses associated with recovery following ECMO discontinuation. Considering the substantial economic impact of ECMO therapy, further research is warranted to optimize its cost-effectiveness while maintaining favorable outcomes.

### 4.5. Clinical Implications

These findings have important implications for patient selection and clinical decision-making. The identification of strong pre-ECMO predictors, including organ dysfunction and ASA classification, may guide clinicians in selecting candidates who are more likely to benefit from VA-ECMO. Moreover, the high incidence of complications underscores the need for proactive strategies to mitigate bleeding, renal dysfunction, and neurological injuries. Enhanced post-ECMO care protocols and multidisciplinary approaches may improve survival outcomes.

### 4.6. Future Directions

Although this study provides valuable insights, further prospective research is needed to refine survival prediction models and examine long-term outcomes beyond hospital discharge. In addition, studies incorporating biomarkers, advanced hemodynamic parameters, and machine learning models may enhance risk stratification and improve clinical decision-making in patients undergoing VA-ECMO.

### 4.7. Limitations

This study presents some limitations. First, as this was a retrospective, single-center study, the findings may not be generalizable to other institutions with different patient populations or ECMO management protocols. Second, although multivariate analysis was performed to adjust for confounding factors, unmeasured variables may have influenced the survival outcomes. Third, the sample size, particularly for certain subgroups, was relatively small, potentially limiting the statistical power to detect small differences. Fourth, we did not distinguish between deaths occurring during ECMO support and those occurring after successful weaning, as detailed timing data were not consistently available in the retrospective records, and stratification would have further reduced statistical power. In addition, this study did not assess long-term outcomes beyond in-hospital survival, which could have provided a more comprehensive understanding of post-ECMO prognosis. Future multicenter prospective studies with larger sample sizes, long-term follow-up, and detailed time-stamped data are warranted to validate these findings, explore mortality timing patterns, and improve ECMO patient selection and management strategies.

## 5. Conclusions

We highlighted the significant factors associated with survival in patients undergoing VA-ECMO, including the type of ECMO approach, preexisting organ dysfunction, and ASA classification. Peripheral VA-ECMO was associated with improved survival, whereas liver dysfunction and renal dysfunction were strong predictors of mortality. These findings underscore the importance of careful patient selection and pre-ECMO risk stratification for optimizing outcomes. Despite its limitations, this study provides valuable insights that may aid in clinical decision-making and patient management. As this was a single-center retrospective analysis, further studies are warranted to explore the broader applicability of these findings across multiple institutions. Future research should focus on refining prediction models and examining the long-term survival and quality of life of patients undergoing VA-ECMO to further enhance care strategies. By addressing these challenges, this study provides valuable data to improve patient selection, refine risk stratification models, and optimize post-ECMO management.

## Figures and Tables

**Table 1:** Baseline characteristics of patients undergoing VA-ECMO, stratified by in-hospital survival status.

Variables	Survivors (*n* = 50)	Nonsurvivors (*n* = 73)	Total (*N* = 123)	*p* value
VA-ECMO approach				0.198
Central	2 (4)	8 (11)	10 (8.1)	
Peripheral	48 (96)	65 (89)	113 (91.9)	
Anesthesia type				0.071
Local	4 (8)	16 (21.9)	20 (16.3)	
General	46 (92)	57 (78.1)	103 (83.7)	
Sex (male)	32 (64)	47 (64.4)	79 (64.2)	1
Age (years); median (IQR)	56 (39, 63)	59 (45, 68)	58 (43, 67)	0.099
Body mass index (kg/m^2^); mean (SD)	22.9 (3.7)	23.6 (4.3)	23.3 (4)	0.363
Systolic blood pressure (SBP; mmHg); mean (SD)	116.7 (31.1)	98.5 (25.7)	106.4 (29.4)	0.002⁣^∗^
Diastolic blood pressure (DBP; mmHg); median (IQR)	60 (50, 80)	55 (45, 65)	60 (46.2, 70)	0.018⁣^∗^
Mean arterial pressure (MAP; mmHg); median (IQR)	75.5 (66.5, 97.8)	69 (55.5, 79.8)	73 (60, 88.8)	0.01⁣^∗^
Heart rate (HR; bpm); mean (SD)	104.1 (30)	103.7 (26.3)	103.9 (27.8)	0.946
Respiratory rate (RR; bpm); median (IQR)	15 (12.2, 23)	16 (14, 20)	16 (13.8, 20)	0.946
SpO_2_ (%); median (IQR)	100 (93.8, 100)	98 (88, 100)	99.5 (89.5, 100)	0.336
Body temperature (Celsius); mean (SD)	36.7 (1.4)	36.5 (1.4)	36.6 (1.4)	0.48
Glasgow Coma Scale; median (IQR)	10 (5.2, 15)	7 (2, 10)	9 (2, 15)	0.013⁣^∗^
Comorbidities				
Dyslipidemia	8 (16)	21 (28.8)	29 (23.6)	0.155
Hypertension	17 (34)	31 (42.5)	48 (39)	0.449
Diabetes	12 (24)	20 (27.4)	32 (26)	0.832
Atrial fibrillation	12 (24)	13 (17.8)	25 (20.3)	0.542
Chronic kidney disease	4 (8)	6 (8.2)	10 (8.1)	1
Heart failure	10 (20)	19 (26)	29 (23.6)	0.577
Old CVA	7 (14)	9 (12.3)	16 (13)	1
Old MI	13 (26)	14 (19.2)	27 (22)	0.499
ASA classification				0.001⁣^∗^
≤ 3	12 (24)	3 (4.1)	15 (12.2)	
4	23 (46)	31 (42.5)	54 (43.9)	
5	15 (30)	39 (53.4)	54 (43.9)	
Cardiac arrest before ECMO	17 (34)	32 (43.8)	49 (39.8)	0.364
Other organ dysfunction				
Liver dysfunction	13 (26)	42 (57.5)	55 (44.7)	0.001⁣^∗^
Renal dysfunction	15 (30)	41 (56.2)	56 (45.5)	0.007⁣^∗^
Respiratory dysfunction	31 (62)	59 (80.8)	90 (73.2)	0.035⁣^∗^
CNS dysfunction	18 (36)	41 (56.2)	59 (48)	0.044⁣^∗^
Etiology				
Acute coronary syndrome	18 (36)	29 (39.7)	47 (38.2)	0.819
Aortic disease	5 (10)	5 (6.8)	10 (8.1)	0.526
ARDS	1 (2)	3 (4.1)	4 (3.3)	0.645
Cardiomyopathy	3 (6)	6 (8.2)	9 (7.3)	0.737
Congenital heart disease	2 (4)	5 (6.8)	7 (5.7)	0.7
Hypoxic cardiac arrest	4 (8)	4 (5.5)	8 (6.5)	0.714
Massive pulmonary embolism	2 (4)	2 (2.7)	4 (3.3)	1
Myocarditis	4 (8)	6 (8.2)	10 (8.1)	1
Postcardiotomy	21 (42)	21 (28.8)	42 (34.1)	0.185
RV failure	2 (4)	4 (5.5)	6 (4.9)	1
Severe trauma	0 (0)	1 (1.4)	1 (0.8)	1
Valvular heart disease	14 (28)	11 (15.1)	25 (20.3)	0.128
On high-dose vasoactive	34 (68)	66 (90.4)	100 (81.3)	0.004⁣^∗^
On the intraaortic balloon pump	7 (14)	23 (31.5)	30 (24.4)	0.045⁣^∗^
Ventilator and ABG parameters				
PaO_2_/FiO_2_ ratio; median (IQR)	148.7 (92.9, 231.8)	115.3 (70, 260.5)	125 (79.8, 246.2)	0.139
Peak inspiratory pressure (cmH_2_O); median (IQR)	22 (16, 25)	23 (19, 27.8)	23 (17, 26)	0.242
PEEP (cmH_2_O); median (IQR)	5 (5, 5)	5 (5, 8)	5 (5, 7)	0.11
pH; median (IQR)	7.3 (7.2, 7.4)	7.2 (7.1, 7.4)	7.3 (7.1, 7.4)	0.042⁣^∗^
PaCO_2_ (mmHg); median (IQR)	38.9 (31.5, 47)	38.8 (32.8, 51)	38.8 (32.4, 48.2)	0.425
PaO_2_ (mmHg); median (IQR)	103.4 (71.9, 195.2)	95.1 (65.9, 176)	98.6 (68, 177.5)	0.42
HCO_3_^−^ (mmol/L); mean (SD)	19.2 (5.2)	15.7 (6.1)	17.2 (6)	0.001⁣^∗^
Lactate (mmol/L); median (IQR)	3.8 (1.4, 11.9)	8.9 (4.8, 13.9)	7.9 (2.9, 12.9)	0.017⁣^∗^
Decision factors				
Time from intubation to ECMO (days); median (IQR)	0.2 (0.1, 0.7)	0.4 (0.1, 2)	0.2 (0.1, 1)	0.107
Time from intubation to ECMO (category) (days)				0.345
≤ 2	45 (90)	60 (82.2)	105 (85.4)	
> 2	5 (10)	13 (17.8)	18 (14.6)	
Time from ICU admission to ECMO (days); median (IQR)	0.5 (0, 2)	0.3 (0, 2)	0.3 (0, 2)	0.723

*Note:* cmH_2_O, centimeter of water; mmHg, millimeter of mercury; PaO_2_, partial pressure of oxygen; FiO_2_, fraction of inspired oxygen; HCO_3_^−^, bicarbonate; mmol/L, millimole per liter.

Abbreviations: ECMO, extracorporeal membrane oxygenation; ICU, intensive care unit; IQR, interquartile range; PEEP, positive end-expiratory pressure; SD, standard deviation.

^∗^Indicates statistical significance at *p* < 0.05, variables with *p* < 0.05.

**Table 2:** Comparison of cardiac arrest profiles in survivors and nonsurvivors undergoing VA-ECMO.

Variables	Survivors (*n* = 17)	Nonsurvivors (*n* = 32)	Total (*N* = 49)	*p* value
Rhythm				0.508
Asystole	1 (6.7)	4 (13.4)	5 (11.1)	
PEA	4 (26.7)	14 (46.7)	18 (40)	
VF	6 (40)	7 (23.3)	13 (28.9)	
VT	4 (26.7)	5 (16.7)	9 (20)	
Witnessed arrest	17 (100)	30 (93.8)	47 (95.9)	0.537
Time from arrest to CPR (min); median (IQR)	0 (0.0)	0 (0.0)	0 (0.0)	0.709
Time from arrest to ECPR activation (min); mean (SD)	22.2 (20.4)	16.4 (19.5)	18.4 (19.6)	0.505
Time from arrest to ECMO flow (min); mean (SD)	52.3 (22.7)	60.3 (31.8)	57.5 (28.9)	0.381
ROSC	15 (93.8)	24 (75)	39 (81.2)	0.238

Abbreviations: IQR, interquartile range; PEA, pulseless electrical activity; ROSC, return of spontaneous circulation; SD, standard deviation; VF, ventricular fibrillation; VT, ventricular tachycardia.

**Table 3:** Predictors of survival based on univariate and multivariate analyses.

Variables	Univariate analysis		Multivariate analysis	
	Crude OR (95% CI)	Crude *p* value	Adj. OR (95% CI)	*p* value
Approach: peripheral vs. central	3.36 (0.68, 16.69)	0.138	26.44 (1.95, 358.7)	0.014⁣^∗^
Anesthesia: general vs. local	4.91 (1.03, 23.46)	0.046	5.49 (0.89, 33.82)	0.067
DLP	0.7 (0.26, 1.85)	0.47	0.28 (0.07, 1.02)	0.054
Liver dysfunction	0.21 (0.09, 0.5)	< 0.001	0.27 (0.09, 0.79)	0.016⁣^∗^
Renal dysfunction	0.24 (0.1, 0.56)	< 0.001	0.29 (0.1, 0.85)	0.023⁣^∗^
ASA classification: Ref. ≤ 3				
4	0.2 (0.05, 0.81)	0.024	0.11 (0.01, 1.05)	0.056
5	0.11 (0.03, 0.5)	0.004	0.07 (0.01, 0.67)	0.022⁣^∗^

*Note:* DLP, dyslipidemia.

Abbreviations: ASA, American Society of Anesthesiologists; CI, confidence interval; ECMO, extracorporeal membrane oxygenation; MAP, mean arterial pressure; OR, odds ratio.

^∗^Variables with *p* < 0.05.

**Table 4:** Complications related to VA-ECMO.

Variables	Survivors (*n* = 50)	Nonsurvivors (*n* = 73)	Total (*N* = 123)	*p* value
AKI	23 (46)	47 (64.4)	70 (56.9)	0.066
Bleeding	12 (24)	30 (41.1)	42 (34.1)	0.077
Infection	28 (56)	41 (56.2)	69 (56.1)	1
Hemolysis	8 (16)	19 (26)	27 (22)	0.272
Coagulopathy	5 (10)	17 (23.3)	22 (17.9)	0.099
Neurologic complications	9 (18)	30 (41.1)	39 (31.7)	0.012^∗^
Cardiac tamponade	6 (12)	12 (16.4)	18 (14.6)	0.671
Limb ischemia	4 (8)	5 (6.8)	9 (7.3)	1
Bowel ischemia	0 (0)	5 (6.8)	5 (4.1)	0.079
Vessel injury	1 (2)	4 (5.5)	5 (4.1)	0.647
Liver infarction	0 (0)	1 (1.4)	1 (0.8)	1
LV distension	0 (0)	1 (1.4)	1 (0.8)	1
Harlequin syndrome	0 (0)	1 (1.4)	1 (0.8)	1

Abbreviation: LV, left ventricle.

^∗^Variables with *p* < 0.05.

**Table 5:** Expenses and other outcomes.

Variables	Survivors (*n* = 50)	Nonsurvivors (*n* = 73)	Total (*N* = 123)	*p* value
Expenses (THB)				
Total admission expenses	997,563.5 (728,416.5, 1,350,748.8)	696,191 (504,406, 1,124,993)	858,533 (571,388.5, 1,199,172)	0.004^∗^
ECMO expenses	198,377.5 (155,130.2, 283,881)	197,795 (153,100, 290,615)	197,795 (153,683, 289,305)	0.645
Medication expenses	56,844.5 (40,411.5, 115,762.8)	42,070 (20,291, 73,877)	48,379 (26,003.5, 90,833)	0.009⁣^∗^
Nonmedication expenses	42,593 (28,499.8, 61,451.2)	29,736 (14,834, 55,433)	37,316 (19,830.5, 56,352.5)	0.007⁣^∗^
Blood products expenses	68,970 (40,712.5, 144,176.2)	63,160 (22,845, 133,580)	67,140 (30,617.5, 137,075)	0.484
Investigation expenses	119,407.5 (83,700, 164,860.5)	69,725 (42,580, 118,875)	94,800 (55,092.5, 148,995)	< 0.001⁣^∗^
Surgery and anesthesia expenses	152,135 (120,282.5, 242,700)	131,100 (80,040, 244,710)	143,250 (94,057.5, 245,030)	0.075
Other outcomes				
Time on ECMO (days)	3.5 (2, 5)	3 (1, 6)	3 (1, 6)	0.145
Time to discharge/death (days)	28.5 (18, 46.5)	3 (1, 9)	10 (2, 28.5)	< 0.001⁣^∗^

*Note:* THB, Thai Baht.

Abbreviation: ECMO, extracorporeal membrane oxygenation.

^∗^Variables with *p* < 0.05.

## Data Availability

The data that support the findings of this study are available from the corresponding author upon reasonable request.

## Nomenclature

AIC: Akaike information criterion
ASA: American Society of Anesthesiologists
AUC: Area under the curve
CI: Confidence interval
CNS: Central nervous system
DBP: Diastolic blood pressure
ELSO: Extracorporeal Life Support Organization
HR: Heart rate
IQR: Interquartile range
MAP: Mean arterial pressure
OR: Odds ratio
PEA: Pulseless electrical activity
ROC: Receiver operating characteristic
RR: Respiratory rate
SBP: Systolic blood pressure
SD: Standard deviation
VT: Ventricular tachycardia
